# Gambling at Work: A Qualitative Study of Swedish Elite Athletes, Coaches, and Managers

**DOI:** 10.1007/s10899-021-10007-4

**Published:** 2021-02-02

**Authors:** Maria Vinberg, Dan Wetterborg, Pia Enebrink

**Affiliations:** 1 Centre for Psychiatry Research, Department of Clinical Neuroscience, Karolinska Institutet and Stockholm Health Care Services, Stockholm County Council, Norra Stationsgatan 69, SE-113 64 Stockholm, Sweden; 2 Division of Psychology, Department of Clinical Neuroscience, Karolinska Institutet, Stockholm, Sweden

**Keywords:** Athletes gambling, Coaches gambling, Sports betting, Sport policy, Problem gambling

## Abstract

Considering the financial connections between sport and the gambling industry, more should be learned about gambling and problem gambling in this setting. This study explores how male athletes, coaches, and sports managers experience gambling activities and problems in their sports. Interviews were conducted with 30 male elite athletes, coaches, and managers in four sports. The interviews were analysed using content analysis, and the results indicated two main themes: 1) desire for and concerns with money and 2) in the shadow of performance, and three categories. The first main theme emerged as a result of the respondents recurring reference to money as the reason to different actions: It is important to win money,’too little’ or ‘too much’ money is described as reason for gambling, athletes status is affected by money and sponsor money from the gambling companies are considered important. ‘In the shadow of the performance’ captures the reason to and value of performance: The thrill and money are rewards for the gambling performance, everyday sporting life emphasizes performance both in training and matches. Lack of successful performance is perceived as a threat and evokes a fear of being seen as weak and being ejected from the team. This study identifies gambling as normalized within male elite sport. Preventing gambling problems calls for action at all levels of the involved socio-ecological framework. Management, coaches, and athletes need more knowledge of gambling and how to create a sustainable framework to prevent gambling problems.

## Background

### Sport and Gambling

Sport is generally connected to physical activity and health outcomes, but for many, it is also connected to gambling and hopes of winning money. Betting on sport is one way to do this. In some countries, such as Sweden, sports betting has been around for decades, while elsewhere, such as in the USA, it has been legalized only recently (Binde 2014). Knowledge of the economic connections between betting, sponsorship, and advertising as well as testimonies and studies regarding problem gambling among athletes have suggested that athletes and even coaches may be societal groups with a higher risk of gambling problems than the general population.

### Gambling as a Public Health Issue

Research into gambling problems often focuses on individual risk factors and on developing interventions for affected individuals. Globally, the prevalence of problem gambling is 0.4–8%, with the Swedish prevalence being about 0.4% (0.7% for men and 0.2% for women) (Williams et al. 2012; Public Health Agency of Sweden 2016). However, a commonly reported prevalence rate is 2%, a figure derived from merging groups with “moderate-risk gambling” and “problem gambling”. These labels describe individuals experiencing some adverse consequences of their gambling and are usually used when addressing populations rather than individuals. “Gambling disorder” is the clinical term used to describe the severest form of this disorder (American Psychiatric Association 2013). Problem gambling is considered a public health problem unequally distributed in society (Rodgers et al. 2015), as studies show that socio-economically weaker groups gamble less but have a higher proportion of gambling problems. Men have a higher proportion of gambling problems than do women and younger men generally have a higher risk of problem gambling (Public Health Agency of Sweden 2016; Billi et al. 2015). Few studies examine problem gambling in specific societal groups, such as interest groups (e.g., sports fans) or occupational groups (e.g., stockbrokers and truck drivers), although some occupations exist in environments where gambling is more salient, such as the gambling industry and elite sports contexts (Hing and Gainsbury 2013). We will return to group-level risk factors below.

### Risk Factors for Gambling Problems

Risk factors for gambling problems can advantageously be described within a socio-ecological framework (Rodgers et al. 2015; Fulu and Miedema 2015; Wardle et al. 2019). This framework captures the multi-level influences on individual behaviour and visualizes the reciprocal effects between the risk factors at different levels.

Divided into four levels, the framework comprises: (1) *individual-level factors*, such as health and psychosocial problems specific to the individual; (2) *family and social network factors* pertaining to relations in one’s closest social circle, such as family members, friends, work colleagues, and teachers; (3) *community-level factors* pertaining to the settings where social relationships occur, such as neighbourhoods, workplaces, and schools; and (4) *societal and commercial-level factors*, i.e., broad societal factors, such as social and cultural norms, as well as the health, economic, educational, and social policies that contribute to economic and/or social inequalities between populations.

Individual risk factors recurrently identified in studies are alcohol abuse, anxiety, depression, impulsivity, and unsafe upbringing (Scholes-Balog et al. 2015; Public Health Agency of Sweden 2013). At the relationship and local community levels, there are risk factors such as being around friends who consider gambling important, having parents with gambling problems, and poor school results (Reith and Dobbie 2011; Dowling et al. 2017; Public Health Agency of Sweden 2013). For example, gambling at work or school has been found to correlate with gambling problems (Public Health Agency of Sweden 2013; Binde 2016). At the societal level, accessibility is a significant factor, with high accessibility being a contributing factor to the normalization of gambling (Thomas et al. 2018; Dowling et al. 2017; Hansen and Rossow 2012). While none of these factors is sufficient to cause problem gambling in isolation, each increases the risk.

At a community level, some occupations might have an elevated risk of problem gambling, as an increased number of risk factors creates higher vulnerability. This could be the case for gambling industry employees. A study by Hing and Gainsbury (2013) found a higher proportion of individuals with gambling problems among gambling industry employees than in society in general; they also found five risk factors relating to problem gambling: workplace motivators, workplace triggers, the influence of colleagues, limited social opportunities, and familiarity with and interest in gambling. These risk factors represent several levels in the socio-ecological model.

### Risk Factors and Problem Gambling in Sport

Several structural and cultural aspects of sport likely provide a good basis for gambling even if the sports environment differs not least in the money involved in each sport and in structural differences, such as team versus individual sports. Some characteristics of the sports environment are potential risk factors for problem gambling: male sport generally involves young men; high competitiveness could generate increased gambling; having gambling companies as sponsors might increase awareness of and thus the frequency of gambling; and sensation-seeking, found in some sports, is sometimes described as a potential risk factor (Grall-Bronnec et al. 2016; Weiss and Loubier 2008).

Only one recent qualitative study examines gambling in the sports context. Lim et al. (2016) interviewed British male football players, and the athletes identified factors such as high salaries, considerable leisure time, and the influence of older teammates as influencing gambling and problem gambling. They emphasized that social factors were important both to starting and continuing gambling:

Many of our interviewees not only believed that participation in gambling activities was good for team spirit but were also aware that immersion into a gambling-rich environment tended to normalise heavy betting practices, making this seem like “the right thing to do”. (Lim et al. 2016, p. 32).

Studies of the prevalence rate of problem gambling among athletes and others in the sports sector are scarce and generally difficult to compare for methodological reasons. In the USA, these studies mainly cover college athletes, reporting prevalence rates between 4.3% (males) and 0.4% (females) (Ellenbogen et al. 2008; Nelson et al. 2007). In Europe, tree different studies showed a prevalence of problem gambling between 6 and 14% among male athletes (Grall-Bronnec et al. 2016; Håkansson et al. 2018; Wardle and Gibbons 2014). Though, they used different timeframe and screening instrument. The British report examining footballers and cricketers (Wardle & Gibbons, 2014) evoked some concern in the Professional Players Federation, as the detected prevalences of 6% problem gambling and 14% moderate-risk gambling far exceeded those of the equivalent group in the general population. The results also showed that the prevalence of gambling problems was higher among low-income than high-income athletes, and that 31% of respondents felt that gambling companies encouraged sportspeople to bet.

Athletes are described as competitive and seeking thrills—or, as it was called by Lim et al. (2016)—“a buzz”. In earlier studies, Weiss and Loubier (2008, 2010) found that former athletes were more likely to have gambling problems than were current athletes and non-athletes, and former athletes were also most likely to gamble on skill-based games. The assumption was that former athletes would have a higher incidence of gambling problems. This is in line with Curry and Jiobu (1995) theory that athletes internalize their competitiveness during their sporting careers, but that this might not surface until after some athletes’ active days are past. Sports betting, another type of perceived skill-based gambling, would then replace the competition formerly provided by sport.

Betting, together with advertising and sponsorship, are three areas where sport is connected to the gambling industry. In many countries, the gambling industry accrues large revenues through sports betting. Sports associations and sports clubs, in turn, benefit from gambling industry sponsorship, and in Europe, advertising revenues from regulated gambling companies have become a significant source of sports funding (EGBA 2019; Lopez-Gonzalez and Tulloch 2015; Milnes 2018; Suncica et al. 2018). Some of these revenues are from athletes’ marketing of gambling products, which has taken a new path in Sweden, where athletes co-own and market gambling companies (Bethard 2018).

### The Previous and Present Study

This study derives from a prior study, conducted by the first author and others (Vinberg et al. 2020), targeting elite athletes and their coaches in four sports in Sweden (i.e., football, ice hockey, floorball, and basketball). The previous study explored the prevalence of gambling and being “at risk of gambling problems”, and the relations between being “at risk of gambling problems” and attitudes towards gambling, experiences of gambling, and individual and demographic factors. Overall, 2% of female athletes and 13% of male athletes were classified as at risk of gambling problems; moreover, the prevalence was higher among coaches of male teams (7%) than among coaches of female teams (3%). The present study aims to follow up on Study I by examining factors within the sport the interviewees experience contribute to whether they themselves and others, gamble or do not gamble, as well as examining how the interviewees perceive the sports clubs’ view and relationship to gambling.

## Method

### Recruitment Process and Sample

Men playing in the highest national league clubs in Sweden in one of three sports, i.e., ice hockey, football, or basketball, were included. Ice hockey and football are major public sports in Sweden that often appear on betting sites. Elite players of these sports are full-time professional athletes and seem to have a higher prevalence of problem gambling than do age-matched population controls. Basketball is a less popular sport in Sweden and less commonly betted on, and the athletes are often semi-professionals. These sports were chosen to enable a broad depiction of different experiences of gambling and problem gambling.

The sample consists of male athletes, coaches, sports managers, and CEOs of male sports clubs, all active at the elite level. This selection was made because of the high proportion of problem gamblers in male elite sport. By focusing resources on male sports, it was possible to reach more respondents in each sport.

In the study, we sampled nine managers at different organizational levels, three coaches, and 19 athletes, all men, representing 17 sports clubs (Table 1). The respondents were recruited from large, medium-sized, and small Swedish cities to include different types of associations, i.e., small and large, resource rich and financially weaker. In recruiting the respondents, we strove for maximum variation. The respondents were recruited to capture potentially different perspectives on gambling, depending on the interviewees’ positions in their clubs. One’s work position in the club might bring knowledge of different processes, for example, policy decisions, as well as different opinions about specific issues. Various recruitment and data collection strategies were employed: direct contact with managers and coaches at some sports clubs, contact via the sports union, and snowball sampling among athletes. The interviews were conducted between April 2018 and November 2018.

**Table 1:** Characteristics of the respondents (in colour) (Color Table online)

Function	Football	Ice hockey	Basketball	Age, years
Managers	2	3	3	35–51
Coaches	1	1	1	34–49
Athletes	7	7	5	20–34

### Interview Guide

A semi-structured interview guide based on findings from previous studies was designed for the study. Each topic was covered in the interviews and probed to varying degrees. The questions were: Please tell us what comes up in your mind when you hear of gambling and sports? How would you describe your own gambling? How would you describe other people's gambling; others in the team and / or in other teams you have been in? What do you think are the main reasons why others in your team or in your previous teams (in which you have been playing) gambles? Please, describe how you think the sport club views gambling?

### Procedure

Each respondent was interviewed face to face by the first author or a research assistant. All interviews, except two, were conducted in the city where the respondents were employed. After obtaining informed consent, we initiated the semi-structured interviews, which lasted 30–120 min. All interviews were digitally recorded and transcribed verbatim. Immediately following each interview, the first author removed all identifying information from the record; the first author, research assistant B, and an authorized transcription agency then transcribed the material.

### Analysis

We conducted a content analysis of the transcripts. The interviews with athletes were analysed separately from those with coaches and managers to ensure that any differences between the groups would be detected. The first author read the interviews several times, while the second and third authors each read half of the material. The authors pre-coded the read material and met several times to discuss the categories to include in the first version of a coding manual. After creating a manual, all three authors’ coded two interviews by dividing the interviews into meaning units, which were condensed and coded. The authors met again to compare and discuss the various codes and categories. After alternating between the raw text, codes, and categories, the manual was slightly revised. (Graneheim and Lundman 2004). The codes were later compared based on differences and similarities and sorted into three categories with three, three, and two sub-categories, respectively and also creating two overarching themes (Table 2).

**Table 2:**
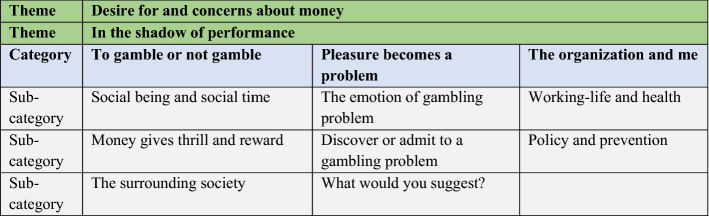
The themes, categories, and sub-categories (in colour)

When analysing the interviews with the coaches and managers, the authors used the same manual when coding and categorizing the first two interviews. The authors then compared and discussed the relevance of the sub-categories and categories. Some of the sub-categories were then slightly revised. The quotations have been slightly edited to maintain respondent anonymity, and/or for quotations to be understandable in English. In the quotations, the group of managers and coaches are collided in order to secure anonymity and then referred to as MC and IP number. The athletes are only referred to with a IP number.

## Results

### Themes

The result yielded two themes and three categories (Table 2). The components of the themes are found in all three categories.

The first theme, “Desire for and concerns about money”, points to recurring references to money, whether as a motivation to gamble, an interpretation of gambling problems, or reason for accepting gambling company sponsorship. It was not the scarcity of money that was a driving force, but rather a mixture of money as a reward and as something that strengthens one’s position, and this was found both at the individual level and when talking about the club. The second theme, “In the shadow of performance”, captures the underlying feeling of constant evaluation and need to perform. Not only the athletes were aware of this, but the coaches were also part of this world of evaluation and performance. Even gambling was partly seen as performance: “being good at gambling” was performance and to win money was to succeed, i.e., perform well. The lack of measurable success was a security threat, as one might lose one’s job, the club might lose matches, or sponsors might leave. Having to admit to a gambling problem was often described as a security threat and a collapse of the idea of being good at gambling.

### To Gamble or not Gamble

The most common respondents’ explanations of why they gamble were “the thrill” and “the importance of winning money”. However, in line with previous studies of social networks, the social context was found to be important in generating and sustaining gambling (Hing and Gainsbury 2013; Reith and Dobbie 2011). Descriptions that capture the social context constitute the basis of the results, which is why we begin by depicting them.

### Social Beings and Social Time

Regardless of age and position, respondents said that gambling had always been part of their sports environment. Some respondents did not gamble themselves, others gambled and had done so for many years, while still others no longer gambled.

The gambling sometimes started with teammates talking about money and the conversations often started with the same group of athletes. I happened that the conversations about gambling sometimes ceased when those athletes moved to another club. The respondents, both those who were young athletes at the time of the interview and those looking back on when they were younger, talked about being inspired by older athletes and those who seemed to win a lot of money:

But if someone comes into the locker room and says he won 500,000 or has bet 50,000 on a match. Then you listen carefully: What match did you play? … and so on. And so it happens over a long period. Obviously, you may think at some point, “Damn, I’ll just try it too”. (IP 7, 24 years).

The talk and sometimes the planning of gambling were part of solidifying a community. The gamblers mainly talked about what to bet on (e.g., horses and various teams) or how much money they would bet and might win, with the focus being on profits. Losses did not occupy much time in the conversation:

But then, there is something to talk about in the dressing room as well. People talk about what to bet on, how to do it, how to bet … (IP 27, 26 years).

There were few distinct differences in how gambling was described by the athletes compared with the coaches/managers. The managers of the larger, wealthier clubs described less distress about the athletes’ gambling patterns and raised no concerns about any other staff member’s gambling habits. Not many athletes in the current study commented on the gambling of the coaches, although it happened:

I think coaches also gamble quite a lot, at least that’s my experience. I have heard many people talk about it. They mention no sums—nothing like that—just that they talk about gambling. You actually get a little shocked, as you didn’t think that was the case. You think they have grown older and understand a little, that there is no meaning to it, really. (IP 2, 26 years).

All respondents experienced that athletes had a relatively large amount of free time. For young people who did not have family, afternoons and evenings were often free, sometimes creating the feeling that time must be “killed”. Gambling become a way to give color to leisure time described as somewhat monotonous. If free time increased, for example, when athletes changed sports clubs and moved to a new town, their gambling might increase:

I would say that I play a little more now since I’ve come to X than I did in Y. … In the beginning, there was a lot of free time and there was nothing to do [here]. (IP 11, 21 years).

Journeys in connection with matches were recurrently described in terms of “time to kill” But also a sociable time. Plenty of hours were spent travelling on the bus, and how to spend that time varied little: Netflix, sleeping, listening to music, gaming, and gambling seemed to be the most common features of such travelling. Some sports clubs have two-floor buses, with the athletes using the upstairs and the coaches the downstairs. It was mainly on the bus that gambling became a shared activity of the athletes. This activity can be difficult to handle, even if there is no formal pressure to participate:

It’s hard to stop when you sit with a bunch [of guys] and gamble at the same time. You come down to zero and watch when others are gambling, then it’s really hard just to give up. (IP 2, 26 years).

One athlete described the time on the bus as the reason why he resumed gambling after refraining from it for some time. Lack of activities and gambling teammates made it seem appropriate to start gambling again.

### Money Gives Thrill and Reward

There is too much or too little money. High salaries and the pursuit of money are factors that athletes, coaches, and management recurrently cite as both as career-driving forces and as risk factors for gambling. The respondents’ earnings differed considerably, but the respondents more or less agreed that higher salaries generated more gambling. A high salary gives one a better opportunity to gamble because one’s surplus is larger. Several respondents described how the stakes increased as incomes increased, or when they became used to high salaries:

So, when I go to the casino with 25,000 plus and then don’t come out with it [i.e., the money], I don’t think it affects me. (IP 10, 28 years).

The same argument was used to explain why female athletes have a much lower prevalence of at-risk gambling than do the males: they don’t have high enough salaries to gamble, nor can they expect to earn them later in their sports careers. However, the respondents reckoned that if salaries increased, gambling might increase in female sport.

“Money at a young age” was commonly described as a risk factor for gambling. Coaches/managers noted that the athletes were relatively young when they first received their high salaries. The young athletes have low fixed expenses but high salaries, making the stakes rise very quickly without immediate consequences.

The idea of fast money was recurrently articulated. Sometimes, in a desire for status, money provides opportunities to consume things and impress others, giving access to contacts, parties, and girls. However, the money won was also a sign that one was good at gambling. The money won from playing the horses, sports betting, or poker became evidence of skill:

I know about it. I can sit and watch a match and check my ability, how good I am at betting. (IP 10 28 years).

The skills are important but were not described as a sufficient reason for gambling; rather, the money is the final goal, the reward and also what gave the thrill. Both regular and occasional gamblers described it as important to win money. Regardless of whether one needs more money or whether the winnings are low, the actual money is an important motivator and a reward.

The elite athletes were described by the coaches, managers, and one another in words such as “reward junkies”, “extreme competitors”, and “results oriented”. Part from money, many of the explanations for gambling concerned “the high” or “the thrill” of winning. The thrill is the reward for success:

It is certainly the thrill we talked about earlier. As an athlete, it is important to get the thrill you get from winning a match. (IP 6, 25 years).

Many respondents bet on sports, and one stated reason for doing so was to increase the thrill. While some respondents reported a need to feel excited when watching a match, others said it was not required. For some, the reactions to the betting outcome were similar to the reactions after having played a match:

If you win, the thrill comes, and if you lose, you do what is expected after a match loss – you get up and take revenge. And these competitive moments both give a thrill and also give some kind of feedback – “Have I done right, have I done wrong?” (IP 16, 45 years, MC).

The thrills one gets from gambling were also used as an escape and relief from setbacks in training, dissipating negative thoughts and creating energy through an “adrenaline kick”.

### The Surrounding Society

Increased availability is a problematic issue mentioned by some respondents, but mainly by the coaches/managers. First came online betting and the possibility of betting on one’s computer; then came mobile betting, and accessibility was unlimited.

All respondents had opinions on gambling marketing. The absolute majority of both athletes and coaches/managers expressed considerable frustration and annoyance at gambling advertising: the amount of such advertising was irritating, and there was the question of whether or not it was harmful. Regarding gambling advertising, many believed that it probably affected the number of people attracted to gambling, as the advertising made it difficult to forget about gambling.

A few respondents noted that it was probably difficult to stop gambling when surrounded by intrusive advertising. Another issue was that some athletes advertise gambling, which some saw as very problematic. This opinion was repeatedly articulated by the athletes, but was just briefly mentioned by one coach/manager. Athletes as horse owners represented another form of marketing, and were mentioned as a trigger to bet on horses. The combination of sponsorship by gambling companies and athletes promoting gambling in advertising illustrated the pervasive influence of gambling companies:

The team is sponsored [by a gambling company] and it is so obvious that you notice it very often. … Obviously, the question arises once again: “Oh, should I start doing that? Zlatan [Ibrahimović] is in the commercials – he thinks it’s a good thing [to gamble] and is my idol. Well, I should register there”. There is an awful lot of advertising for gambling, and it is clear that it is not good for my age group. (IP 18, 20 years).

Two of the three participating sports associations, for football and ice hockey, have gambling companies as sponsors, and several sports clubs have sponsorship agreements with gambling companies. One respondent described a situation in which an athlete with gambling problems was asked to come to the casino to represent the team at an event. The situation could have been avoided, as it was provocative and stressful for both the athlete and his friends who knew about it. Representation was seen as among the athletes’ duties but also as a form of entertainment.

Do you go there [i.e., to the horse races], so … 20% of those sitting in the stands are [sports] guys as well. That’s it – it’s accepted as hell to gamble. (IP 8, 26 years).

The respondents expressed gratitude for the revenues from the gambling companies and unwillingness to forgo that money. However, the choice of sponsors seemed to be a latent issue. The athletes were happy to raise the matter with the clubs and the sports associations, and managers/coaches were convinced that gambling company sponsorship was necessary and beneficial, or were ambivalent about how the issue ought to be handled. One coach described a meeting between ice hockey and football coaches in which they suddenly started discussing gambling company sponsorship. He described this as a sign of concern about the situation. However, the managers generally expressed no concern that the athletes or themselves might become more prone to gambling problems through having gambling companies as partners. The coaches and management differed, in that management was less likely to problematize the issue.

### Pleasure Becomes a Problem

#### The Emotions of Gambling Problems

The respondents who gamble described it as essentially amusing and entertaining. However, several recalled periods when they did not feel good about gambling or described what it was like for others who ended up gambling destructively. Those who reflected on their gambling often did so based on their relationship dynamics, for example, their partner might complain, or they felt too occupied by thoughts of gambling and paid their loved ones too little attention. Still, monetary losses were the main reason for worries about gambling:

A month ago, maybe, I felt like this: “I’m gambling too much, it’s not good”. So, I went back and checked all my accounts, all the withdrawals and deposits. I concluded that in three years I had lost 50 thousand. I don’t know whether that might be considered a gambling addiction. (IP 8, 26 years).

One coach said that he met many young athletes with gambling problems. In general, they were ashamed and blamed themselves:

The guys I talk with who have problems, they call themselves idiots. Their self-image is so bad it can’t get worse. Everyone says almost the same thing: It would have been better to have a drug problem, then you at least would have been addicted to something. (IP 4, 41 years, MC).

### Discover or Admit to a Gambling Problem

Several respondents said that the most important thing was to have control over their finances when gambling; for example, they kept cash records, checked their logs, or sat down and counted their expenses. However, the problems could begin with losing control of finances, and then starting to gamble to restore one’s financial position.

Everyone agreed that it was difficult to discover or assess whether a person has a gambling problem. If they were to ask most were doubtful as to whether they would get an honest answer:

Then, it is also sensitive to ask the question and … People can take offence if you say, like, “How are you, do you have a problem?” It is not easy, either, to open up and just throw it out. But I think you can always ask, at least it’s not bad. (IP 29, 20 years).

The first explicit sign was “when the person starts to borrow money”, but as the respondents also noted, by that point the gambling problem had probably been going on for a while.

Some respondents asked how to relate to a person who gambles a lot but does not seem to feel bad about it. Some considered “gambling often and with too much money” an early sign of “addiction”, though addiction did not seem to be synonymous with “problem”. Some respondents believed that as long as the person did not apparently feel bad about gambling, it could not be considered a problem. It evoked suspicion, however, when gamblers talked only about “profits” and not at all about losses. Everyone knows, the respondents said, that one loses too—the question is just “how much”.

Most athletes said that teammates were likely to suspect or get to know if someone is feeling bad. Both athletes and coaches were clearly aware of their teammates’ general health, but usually relatives or spouses were the first to discover gambling problems:

Then he became greyer, worse, and finally it came out that his partner had found a lot of debt-collection notices at home and thought that this was impossible for a man who earns (X)00,000 in salary a month – you hardly need debt collection. Then she started investigating this, and it turned out that he had wasted all the money they had and borrowed nearly (X)00,000 to finance his gambling. (IP 4, 41 years, MC).

Many said that it was common to hide larger problems, such as gambling problems, from the coaches. The coaches and sports managers confirmed this, saying that even if they suspected that something was wrong, it might take several conversations before the person would open up. Also, telling others about one’s own gambling problems was considered difficult. Some of the younger respondents thought it would be possible to tell teammates if they had a problem, but they would rather talk to their parents or girlfriend. Others described it as difficult and relatively uncommon to talk about sensitive problems in the locker room. On the other hand, gambling problems were considered somewhat easier to talk about than mental illness. There was a fear of being seen as weak, as this might affect their position in the team. Having gambling problems was often described as being stupid rather than weak:

Admitting to gambling addiction can be tough and hard, I think. Well, it’s nothing anyone wants to do … But if you want to look at it purely career-wise, I still think it is more difficult to admit that you feel crap mentally than having problems with gambling, even though it is obviously very closely related. … It is even bad in front of teammates, but even more for the club management. (IP1, 28 years).

### What Would you Suggest?

The respondents were asked what the individual could do to prevent gambling problems.

One respondent said that even though he appreciated gambling and saw it as fun, he no longer initiated gambling with his teammates. Some respondents said they would like to talk more about the downsides of gambling, as there were rarely any critical questions or discussions of gambling among the athletes or in interactions with coaches. If this happened, it might ultimately be possible to change attitudes to gambling, some argued:

It is very uncommon to have reservations, instead you laugh at it: “Haha, you lost 5,000 SEK” – a little bit like that. Everything that has to do with gambling you consider more like “fun”. I think we must get rid of that. (IP 2, 26 years).

Several respondents had found ways to reduce or stop gambling. Some said that their gambling decreased when children and family life made it more difficult to have time to gamble. One respondent felt that he should not gamble for ethical reasons, because gambling companies and gambling created so much trouble for others, but thought that this was too difficult and big a step to take. Others decided that not gambling at all was better than risking being at the borderline of gambling problems.

### The Organization and me

#### Work-life and Health

The spontaneous response of the respondents was that their clubs would help athletes with problems, whether with gambling or anything else. However, all respondents were aware that it is ultimately their performance during training or competition that is crucial. Performance is always in focus, and the feeling of being constantly assessed and valued characterized the athletes’ life choices and self-image. Coaches and managers often returned to descriptions of the centrality of performance.

Sports at this level have been professionalized. All interviewed coaches and managers agreed that the sports milieu is more in line with other workplaces than it was 10–15 years ago, but that more can be done they add. However, they say, the question is what the sport club get back by investing more resources. Everyone, both athletes and coaches/managers, was aware that employment agreements make it possible to eliminate underperforming athletes and coaches. Athlete and coach rotation was seen as part of the business, affecting how much one would want to expose oneself as an individual. Mental health problems can be perceived as weaknesses, creating fear of being traded or removed from the team. On the other hand, denying having a problem can also be problematic:

Say that we have a player with a gambling addiction. We discuss this with the player and he just denies it – then I can tell you that he won’t stay very long in the sports club. (IP 16, 45 years, MC).

All respondents—athletes, coaches, and managers—expressed in different ways that they were aware that personal life affects sports achievement. Despite this, it was described as difficult to trust that club management understood this, as what matters is match results.

### Policies and Prevention

Policies and guidelines are a way to prevent problems and clarify positions, even if they sometimes are insufficient implemented. To start it is notable that the response, in his study, regarding prevention efforts differs with age, particularly among the athletes. Generally, the older the respondent is the more likely he is to suggest some restrictions regarding gambling and believe it would be able to change. Younger respondents are more likely to take the view “it has always been like this—it can’t be changed”.

Most studied clubs had alcohol use guidelines, and all forms of doping were prohibited. It was also common to talk about social media guidelines and how to act as club representatives. However, two subjects reoccurred concerning policy. The first is that “gambling policy” was associated with rules on betting on one’s own sport. It was very unclear to the athletes whether or not one could bet on one’s own league:

No, I don’t have a hundred per cent control, but I know … I know, we can’t bet on our matches. Then I don’t know … I think it may be that we can’t bet on the [league] because it’s our league. (IP 17, 23 years).

Gambling on one’s own matches is prohibited as it could be linked to match-fixing. The police, the National Sports Federation, and to a certain extent the unions have visited the clubs to inform them about match-fixing to prevent this crime.

The second subject is that gambling was not an issue subject to guidelines or structured prevention efforts in any club. Preventive measures consisted of point actions efforts: one respondent talked about a club that banned gambling on the bus to the match, but not on the way home. Another respondent believed that the athletes had been asked not to gamble for money on the bus because it might create incentives for the younger ones to start gambling. One manager said that it was impossible to monitor gambling, but that the athletes should not gamble in the locker room, even though it was difficult to supervise. Respondents’ opinions varied as to the effectiveness of such a gambling policy. Some respondents believed that it would be valuable, but perhaps impossible, to prohibit talk about gambling in the locker room. They thought it would help to shift the focus from money and gambling to something else. A similar situation was described regarding the journeys: the athletes seemed to think that the coaches knew little about what was happening on the bus. They also said that they were not open with the coaches:

No one really talks to the coaches – you are not open about it. [i.e., gambling] with the coaches and such. You are very open within the team, but not with the coaches. (IP 29, 20 years).

Not all the athletes and coaches shared this understanding, and a few athletes and coaches described coaches as more involved.

Investing heavily in prevention efforts may improve results, but not with certainty, some respondents claimed. As there was high mobility between clubs, the club environment can sometimes change rapidly even in the absence of such efforts. Even so, managers stated that, as employers, they are responsible for finding treatments and supporting those with gambling problems, but that it was not yet relevant to formulate guidelines for preventing gambling problems. At the same, time several respondents asked for more information about the warning signs of gambling problems as well as for more information about gambling problems in general. Some clubs have hosted visits by athletes who previously had major gambling problems. The respondents had evidently taken these visits seriously, and the visiting athletes had created respect for the problem.

Some respondents, how had considered the situation, believed that sanctions must be part of preventive measures to restrict gambling if they are to be complied with. There seemed to be some ambiguity about whether, for example, travel time (e.g., bus trips) is considered working or leisure time, and whether that would matter concerning gambling restrictions. It was also deemed important that restrictions should apply to everyone in the sport:

If only our team’s coach says it, I don’t think it would help. If it were something applying to all of Sweden, then it could probably have a greater impact, I think. (IP 2, 26 years).

The only remedy for gambling problems, reported by several respondents, was financial remediation: the club would take the person to the bank and try to rectify his financial problems. If the club saw that the person needed psychological help with his gambling problem, he was referred to outpatient care or even a private CBT-trained psychologist.

Although perceptions of the utility and possibility of gambling policy were diverse, there was one thing that everyone agreed on: start with youth. Regardless of the respondents’ ages, they suggested to start talking about the danger of gambling in high school and youth teams.

## Discussion

### Themes

This study explored how athletes, coaches, and sports managers experience and explain gambling activities and gambling problems in their sports. Two main themes emerged: ‘Desire for and concerns about money’ and ‘In the shadow of performance’. The first theme captures recurring references to money, whether as a motivation for gambling, as creating and enhancing the thrill of gambling, as a reason for accepting sponsorship from gambling companies or as an interpretation of gambling problems. Money seemed to be a symbol of success and a reward for achievement, a way to measure success that was pervasive at both the athlete and manager/coach levels. The theme ‘in the shadow of performance’ captures the underlying feeling of constant evaluation and the need to perform as an athlete. Gambling was sometimes seen as performance: being good at gambling was an achievement and winning money was to succeed, i.e., perform well.

### Social Being and Social Time

Overall, as also found in other studies, gambling is part of everyday life in the investigated elite sports environments (Lim et al. 2016; Moriconi and de Cima 2020). Many athletes described an environment in which “talk about gambling” is common and accepted in connection with training or travel, in line with the results of our previous study (Vinberg et al. 2020). Gambling is generally described as a cohesive factor in the social context of sport and there are few opportunities to problematize it, the normative culture being that gambling is exciting and generally generates income. This description fits previous findings concerning the role of social networks and the process of normalization (Reith and Dobbie 2011; Braams et al. 2014). Team sports demand close cooperation, and coaches and managers said that teams need to thrive to perform well. If gambling is considered to promote cohesion by both athletes and coaches, they are more likely to support than combat it, in turn reinforcing the normative nature of gambling. Russell et al. (2018) showed that higher-risk gamblers have greater interconnectedness in their networks, possibly making it more difficult to reduce or remove the influence of the network. In a professional team sport, this might obstruct preventive efforts, if one does not address the whole group and work with the values that are the basis of normalization.

### To Gamble and not to Gamble

Considering what the respondents identified as reasons for gambling for money, there are factors related to every level of the socio-ecological framework (Wardle et al. 2019). Our results identify three sub-categories of reasons for gambling or not gambling: 1) people as social beings 2) money as rewards and thrill, and 3) the surrounding society.

As a social being, one is influenced by others and the environment. This study has described the social aspect of gambling as a significant factor. Our previous study (Vinberg et al. 2020) found that “talk about gambling during training” was correlated with the risk of gambling problems, highlighting the risk of normalizing gambling at the community level, for example, in sports clubs. This community-level or workplace normalization is reinforced by the increased availability of gambling as well as the amount of gambling advertising, which evoked concern in respondents. The accessibility of gambling as well as gambling advertising concerning sport and featuring well-known sports figures have a normalizing effect on views of gambling (Thomas et al. 2018a; Lopez-Gonzalez et al. 2018). Some athletes were critical of the fact that famous athletes advertise gambling, thinking that it reinforces the image that gambling is harmless, especially given that young athletes look up to their idols.

Sponsorship money is important in elite sport, and many athletes, but few managers, in the study were ambivalent about having gambling companies as sponsors. This ambivalence was prompted by the gambling problems that they all knew existed in sport and by critical discussion throughout Europe as the gambling industry gains dominance as a sports sponsor (Armstrong 2018; BBC 2017; Cook 2019).

The gambling-related factor that most people spontaneously reported was “the thrill” one can get from gambling, which is a big part of its attractiveness (Baudinet and Blaszczynski 2013; Pantalon et al. 2008). This thrill is not specific to athletes, but it is important for the differences in continued gambling between individuals (Baudinet and Blaszczynski 2013). Some respondents who mentioned the thrill as a factor also said that it helped them forget worries (e.g., about not performing adequately in their sport) or counter boredom. One previous study of athletes found that having a high positive urgency score was associated with gambling problems (Grall-Bronnec et al. 2016). This might be related to the description of “the thrill” and could be a factor meriting further research.

In connection with “the thrill”, both managers/coaches and athletes talked about themselves and other elite athletes as “reward junkies” with an “extreme competitive spirit”, described as a strong craving to win, including, in the case of gambling, to win money. The problem with this argument is its loose definition of “competitive spirit”, possibly because “sport” is strongly associated with competition for many individuals (Grindstaff and West 2006). Current studies indicate that the prevalence of gambling and problem gambling among elite athletes differs between genders and countries in the same sport (Wardle and Gibbons 2014; Grall-Bronnec et al. 2016; Vinberg et al. 2020). If the lack of strong competitive spirit is arguably a major factor explaining less gambling and problem gambling among female athletes, we might need a more precise definition of the concept. Weiss and Loubier (2010) Weiss and Loubier (2010) studied the gambling behaviours of current athletes, former athletes, and nonathletes (all men and women with disordered gambling) to explore whether, compared with current athletes and nonathletes, former athletes’ competitive spirit would result in the choice of skill-based gambling. Their hypothesis was that the competitive spirit would manifest itself in former athletes choosing gambling seen as skill based rather than chance based. Their results supported the hypothesis, regardless of gender, but do they point to a manifestation of “competitive spirit”? The authors gave an alternative explanation: “rather than a competitive spirit fueling their high involvement in sports gambling, former athletes simply may be more familiar and feel more comfortable in betting on sport”.

Comparing our results with those of Lim et al. (2016) suggests that “reward” would be interesting to investigate as a prominent factor. Winning money is often described as a kind of reward. Assuming that reward is a major trigger, sports betting would be a more likely choice, as it increases the assumed possibility of winning. In both this study and that of Lim et al. (2016), there are several witnesses to the importance of achievement and of the fear of failing to achieve. Our respondents tended to describe their gambling as sometimes compensating for lack of success in sport. One respondent even claimed that it was “the losers” in the team who talked about gambling the most. This does not explain the previously found differences in gambling prevalence between genders (Vinberg et al. 2020). It could, however, be of interest to look further into how “reward” is conceptualized and operationalized among male and female athletes.

Many respondents associated high salaries with increased gambling, confirming Lim et al. (2016), though assuming that it is a major significant risk factor for problem gambling among these elite athletes might be risky. Our previous study (Vinberg et al. 2020) showed that there is an equally large proportion of gambling and problem gambling in floorball, where salaries are much lower and few athletes are employed full time in the sport. Nor did the level of employment in the sport stand out as significant for the risk of problem gambling (Vinberg et al. 2020). In the current study, some respondents said that they had gambled even when they were earning less money. Perhaps, as found by Lim et al. (2016), those considered to have both high salaries and gambling problems were already gambling in a risky way when they earned less money. When their salaries increased the gambling stakes escalated. As suggested in earlier findings; the increase in bet size might be motivated by increasing chances of bigger wins for hopes of changing luck (Blaszczynski et al. 2008). The thrill and money as a kind of reward are two factors that overlap in the respondents’ descriptions. Free time is also a factor mentioned by both Lim et al. (2016) and our respondents. Then again, having spare time might be a risk factor, but not a decisive factor in this context. There are almost equally high proportions of problem gambling in men’s floorball and basketball, both of which are high-level sports but whose athletes have part- or full-time jobs along with their sports careers (Vinberg et al. 2020). However, our respondents clearly stated that when gambling problems arose, excessive free time was deemed an aggravating factor.

### Pleasure Becomes a Problem

One reason why free time can be a risk factor for increased gambling is its link to the recurring points of stress embedded in sport as a profession (Lim et al. 2016; Roderick 2006). Changing sports clubs and moving to a new town, as well as periods when athletes feel they are performing inadequately are times of transition that some respondents believe can lead to increased gambling. It was previously found that gambling can be used to escape unpleasant feelings or divert thoughts from stressful circumstances, and these findings were confirmed in our interviews (Weatherly et al. 2018; R. T. A. Wood and Griffiths 2007).

All respondents agreed that gambling problems are difficult to detect and define. It might take some time for athletes to open up and disclose their problems, probably for fear of showing weakness and being stigmatized (Lim et al. 2016; Hing et al. 2016). Also, not every club or coach was said to pay attention to mental health issues, making it even harder to report a problem to one’s superiors and employer, a problem documented elsewhere (Rice et al. 2016; Purcell et al. 2019). This brings us to the subject of responsibility.

### The Organization and me

Professionalization and society’s expectations of documentation and policy were well known to the respondents and have also been noted in previous research (Fahlén and Stenling 2016). However, certain sport-specific factors affect the clubs’ motivation to address gambling, and perhaps also mental health, preventively. All respondents were aware that in their sports (most clearly in football and ice hockey) they were interchangeable, i.e., one is no better than one’s last performance in a match. The constant assessment and precise performance requirements that are part of their everyday life are two factors that generated doubts among the athletes concerning whether their sports clubs would want to address preventive issues. This issue also arose among the coaches. There was an underlying suspicion that unless a problem was quickly resolved when discovered, the person involved would be ejected from the organization to “solve the problem”. This engendered a fear of exposure counteracting the possibility of early detection of emerging problems. Earlier findings emphasize the importance of coaches for the organizational climate, both in creating a performance culture of mastery that addresses athlete stress and in creating a supportive attitude towards mental health issues (Rice et al. 2016).

It was difficult for the athletes to reach out to offer help, as receiving help is associated with lost control and often evokes shame (Gulliver et al. 2012; Lim et al. 2016; Kutcher et al. 2016). Responsibility for both support to treatment and preventive work lies with the employer, i.e., the sports club. Policy or law regulates alcohol and substance use, so athletes and coaches know what to expect. Concerning gambling, both policy and structured preventive work are lacking in sports clubs, and it is clear that both athletes and coaches/managers are unsure what they can and should do (Vinberg et al. 2020). For example, is time spent travelling work or leisure time? Can or should it be regulated, or do athletes and coaches have the right to oppose workplace directives during such travel? For a start, some athletes suggested that the clubs should discuss problem gambling more often and provide more information about it. Considering the reported difficulties in understanding when gambling becomes a problem, this must be seen as an adequate suggestion, particularly as health literacy and stigma must be addressed if problem gambling and mental health problems are to be prevented (Gulliver et al. 2012; Moore et al. 2017; S. Wood et al. 2017).

## Strengths and Limitations

This study has several strengths. It largely builds on, deepens, and nuances the findings of a previous study of the prevalence of gambling and gambling problems among elite Swedish athletes (Vinberg et al. 2020). Using a qualitative approach lets us explore issues impossible to address in a quantitative study. Another strength was the sampling of participants from three different sports, while a final strength was obtained by interviewing athletes, coaches, and managers, giving a broad overview of the situation in the sports clubs.

The present findings should be considered in light of certain limitations. The interviews were conducted in Sweden and should be interpreted in this social and legal setting. The participants were of various ages and recruited from different parts of Sweden. As the study was exploratory and the method qualitative, the results do not convey any causal explanations of the trajectories of gambling problem emergence. Some respondents initially focused on match-fixing, which might have affected their general feelings about gambling. A final limitation is that this study looked at male sport only, meaning that there is a need for a similar study in the female sports milieu.

## Conclusions

The present results indicate that gambling is a normalized activity in elite sport. The reasons given for gambling and the gambling-related consequences described by the respondents are factors within a particular socio-ecological framework. The determinants of normalization in this context are factors such as individual “thrill” seeking, workplaces where gambling activities are seen as ordinary, unfettered access to and availability of gambling, and sports sponsorship by gambling companies, making them into apparently “good” partners and responsible actors in society.

Preventing problem gambling calls for activities at all levels in the socio-ecological framework. The issue cannot be addressed simply by pushing responsibility downwards in the hierarchy, leaving coaches and athletes on their own. Club management, as well as the coaches and athletes, need more knowledge of gambling problems and how to build a sustainable framework to prevent them.
